# Supplementary material for: Prevalence and risk factors of frailty in older adults with diabetes: A systematic review and meta-analysis
Source: PLoS One. 2024 Oct 31;19(10):e0309837. doi: 10.1371/journal.pone.0309837 (PMC11527323; doi:10.1371/journal.pone.0309837)
Supplement: S1 Fig — (PDF) [file pone.0309837.s001.pdf]

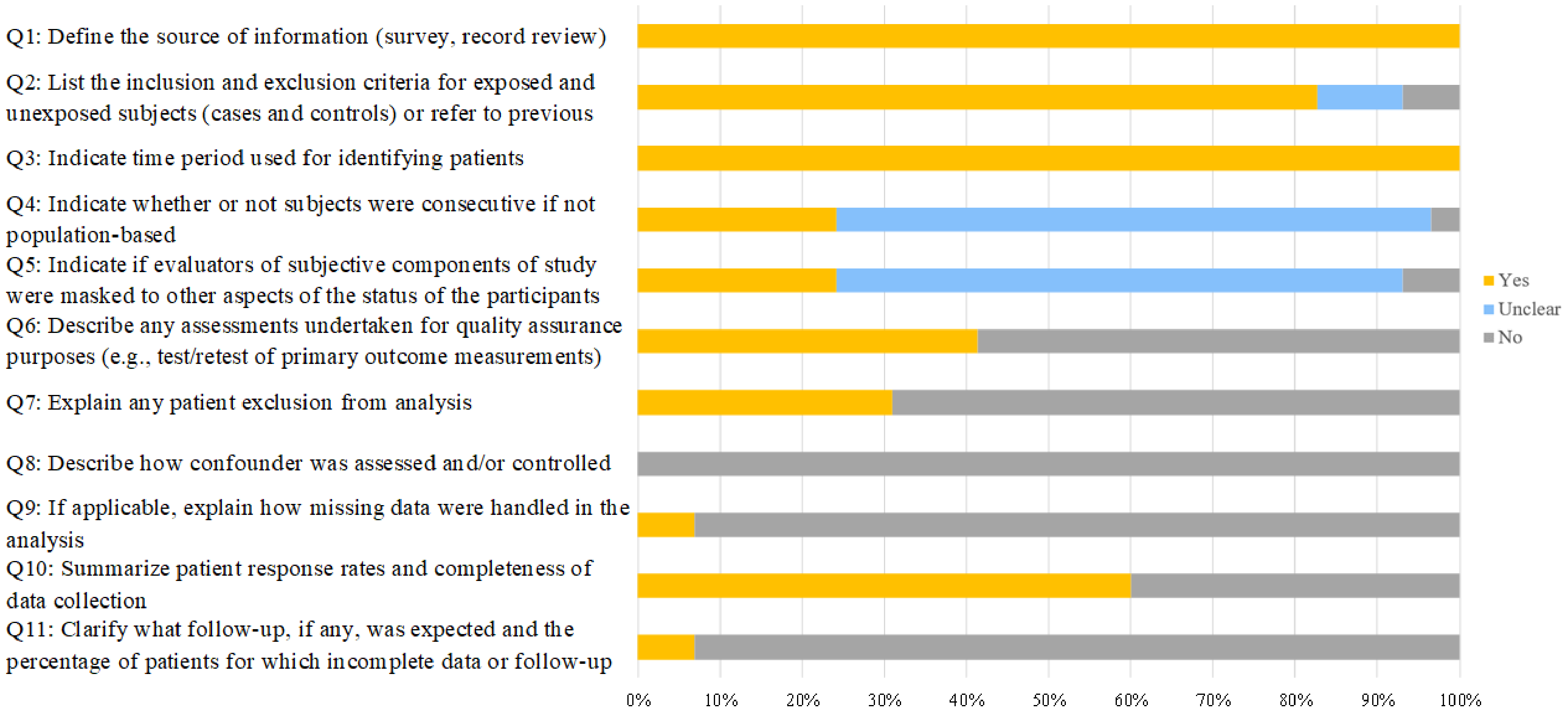
Q1: Define the source of information (survey, record review)
Q2: List the inclusion and exclusion criteria for exposed and unexposed subjects (cases and controls) or refer to previous
Q3: Indicate time period used for identifying patients
Q4: Indicate whether or not subjects were consecutive if not population-based
Q5: Indicate if evaluators of subjective components of study were masked to other aspects of the status of the participants
Q6: Describe any assessments undertaken for quality assurance purposes (e.g., test/retest of primary outcome measurements)
Q7: Explain any patient exclusion from analysis
Q8: Describe how confounder was assessed and/or controlled
Q9: If applicable, explain how missing data were handled in the analysis
Q10: Summarize patient response rates and completeness of data collection
Q11: Clarify what follow-up, if any, was expected and the percentage of patients for which incomplete data or follow-up
0%
10%
20%
30%
40%
50%
60%
70%
80%
90%
100%
Yes
Unclear
No
