# Supplementary material for: Prevalence and risk factors of frailty in older adults with diabetes: A systematic review and meta-analysis
Source: PLoS One. 2024 Oct 31;19(10):e0309837. doi: 10.1371/journal.pone.0309837 (PMC11527323; doi:10.1371/journal.pone.0309837)
Supplement: S2 Fig — (PDF) [file pone.0309837.s002.pdf]

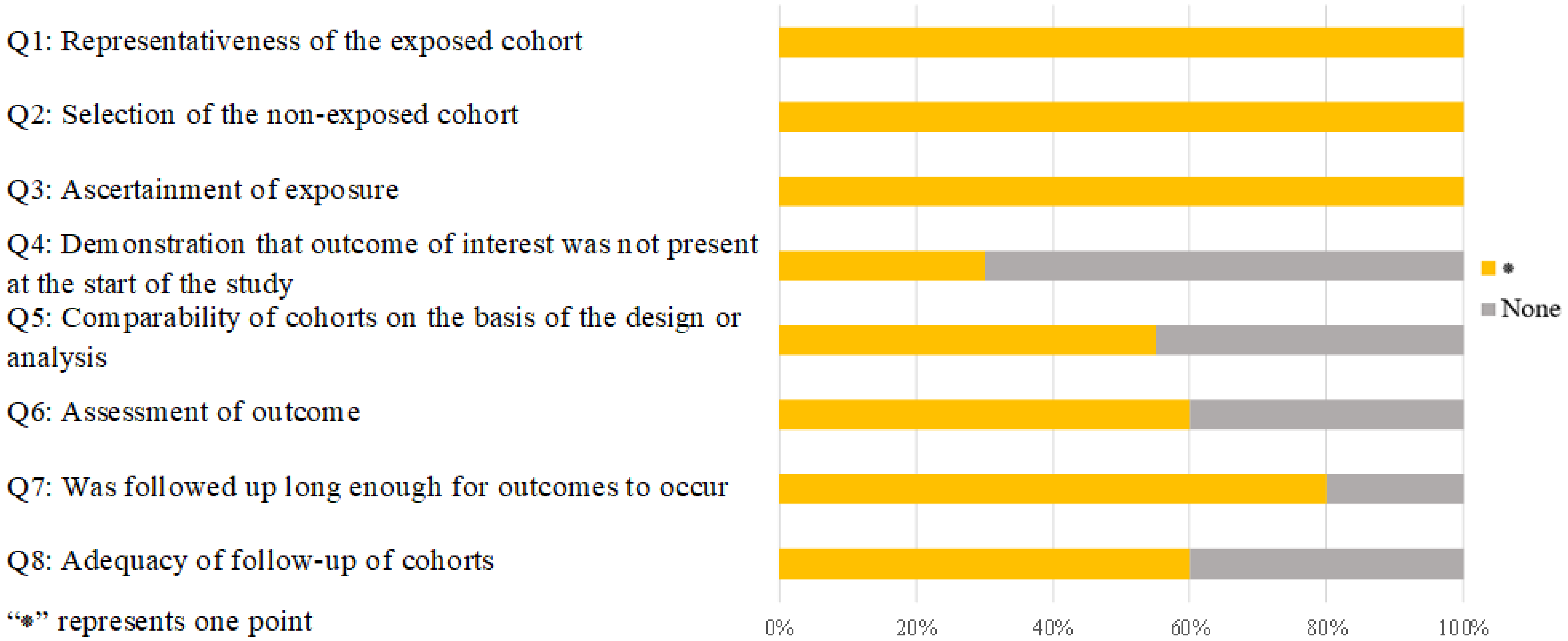
Q1: Representativeness of the exposed cohort
Q2: Selection of the non-exposed cohort
Q3: Ascertainment of exposure
Q4: Demonstration that outcome of interest was not present at the start of the study
Q5: Comparability of cohorts on the basis of the design or analysis
Q6: Assessment of outcome
Q7: Was followed up long enough for outcomes to occur
Q8: Adequacy of follow-up of cohorts
"*" represents one point
0%
20%
40%
60%
80%
100%
*
None
