# Supplementary figures and images for: Prevalence and risk factors of frailty in older adults with diabetes: A systematic review and meta-analysis
Source: PLoS One. 2024 Oct 31;19(10):e0309837. doi: 10.1371/journal.pone.0309837 (PMC11527323; doi:10.1371/journal.pone.0309837)

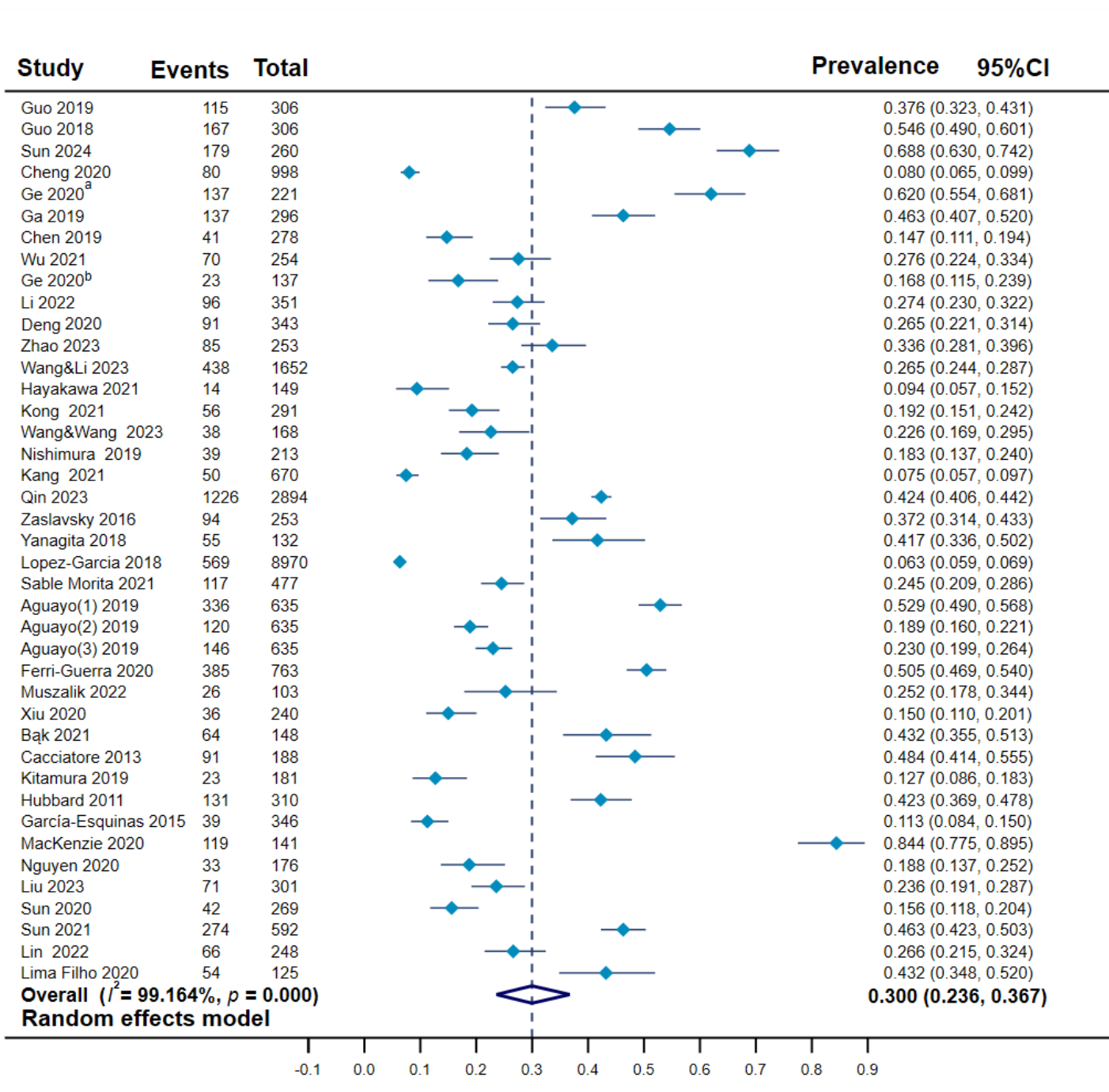

Supplement: S3 Fig — (PDF) [file pone.0309837.s003.pdf]

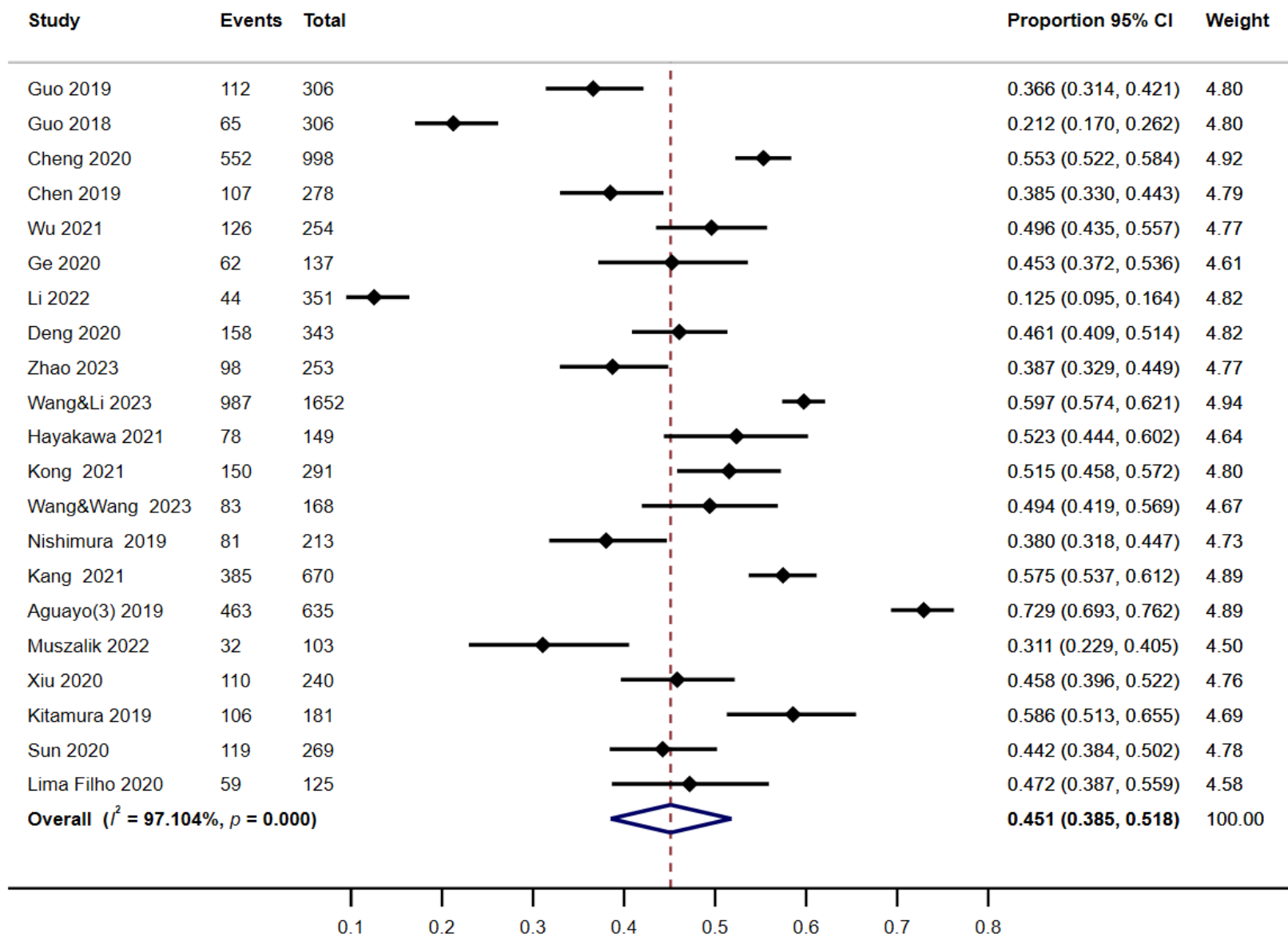

Supplement: S4 Fig — (PDF) [file pone.0309837.s004.pdf]
