# Supplementary material for: Prevalence and risk factors of frailty in older adults with diabetes: A systematic review and meta-analysis
Source: PLoS One. 2024 Oct 31;19(10):e0309837. doi: 10.1371/journal.pone.0309837 (PMC11527323; doi:10.1371/journal.pone.0309837)
Supplement: S5 Fig — (PDF) [file pone.0309837.s005.pdf]

S5 Fig. Meta regression analysis

A)Frailty prevalence and gender (Female %)

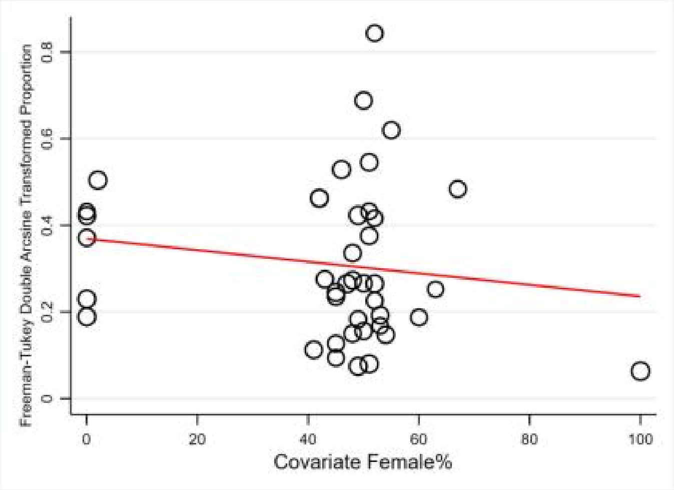

Intercept: 0.369 (95% CI 0.235-0.502), p<0.001; Female %, estimate: -0.133 (95% CI (-0.409) - (0.502)), p >0.1, Adj R2 = 0.20%

B)Frailty prevalence and mean age

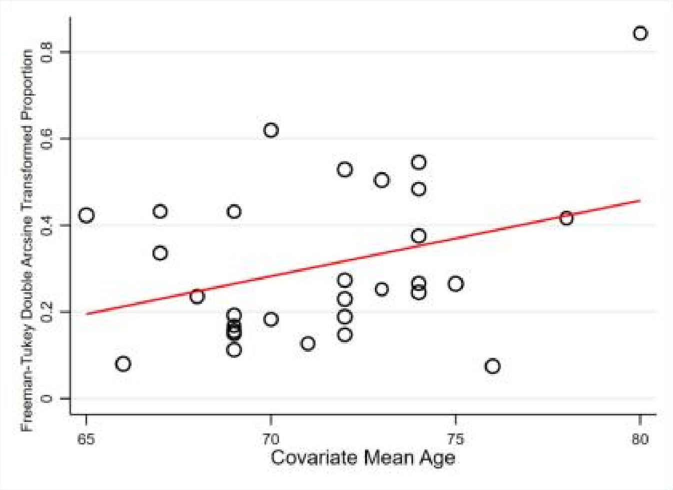

Intercept: -0.942 (95% CI (-2.297)-(0.413)), p>0.1; mean age, estimate: 0.017 (95% CI (-0.001) - (0.036)), p <0.1,Adj R2= 7.51%

C)Frailty prevalence and year of publication

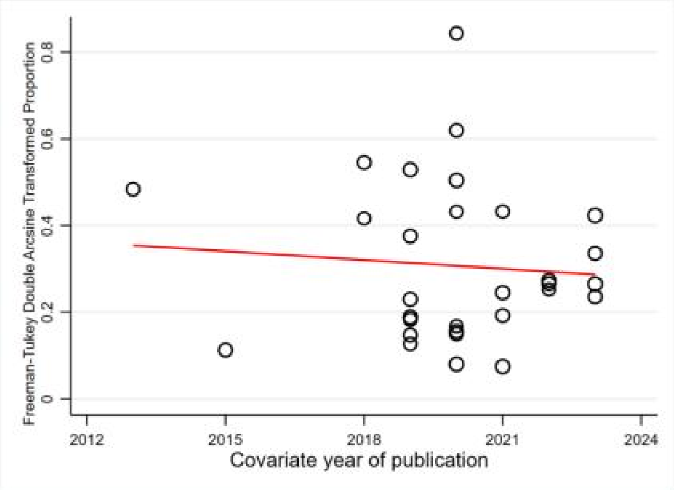

Intercept: 13.936 (95% CI (-49.726)-(77.600)), p>0.1; year of publication, estimate: -0.007 (95% CI (-0.038) - (0.025)), p>0.1, Adj R2 = -3.48%

D)Pre-frailty prevalence and gender (Female %)

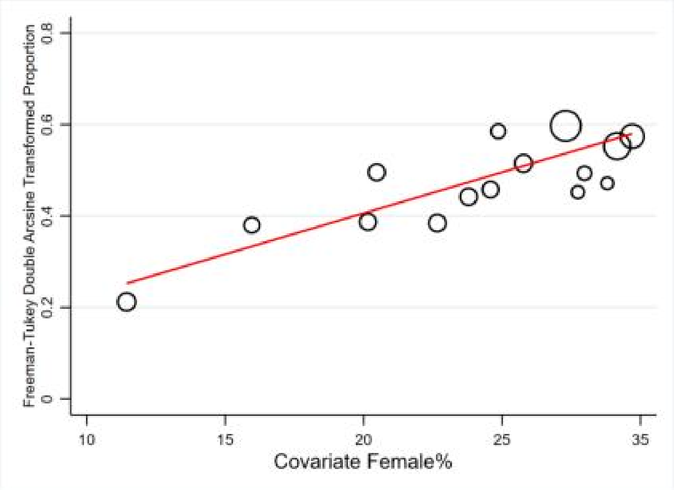

Intercept: 0.047 (95% CI (-0.128-(0.222)), p>0.1; Female %, estimate: 1.794(95% CI 1.106 - 2.483), p<0.001, Adj R2 = 90.23%

E) Pre-frailty prevalence and year of publication

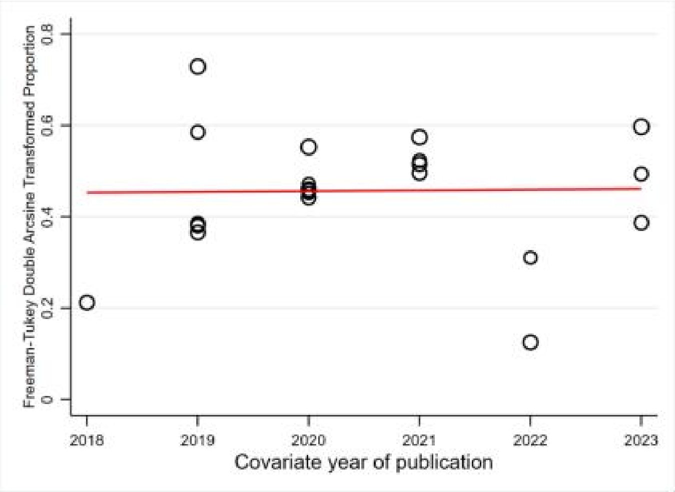

Intercept: -2.990 (95% CI (-95.717)-(89.736)), p>0.1;
year of publication, estimate: 0.002 (95% CI (-0.044) - (0.048)), p>0.1, Adj R2 = -5.87%

F) Pre-frailty prevalence and mean age

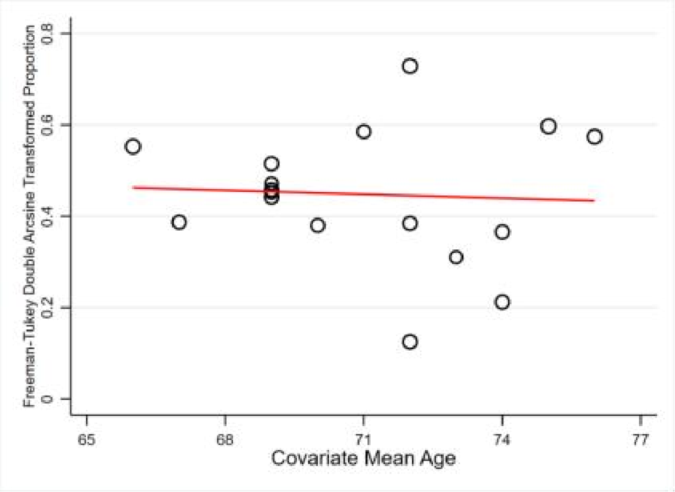

Intercept: 0.652 (95% CI (-1.434)-(2.739)), p>0.1;
mean age, estimate: -0.003 (95% CI (-0.032) - (0.026)), p>0.1,Adj R2= -7.56%
