# Supplementary material for: Prevalence and risk factors of frailty in older adults with diabetes: A systematic review and meta-analysis
Source: PLoS One. 2024 Oct 31;19(10):e0309837. doi: 10.1371/journal.pone.0309837 (PMC11527323; doi:10.1371/journal.pone.0309837)
Supplement: S6 Fig — (PDF) [file pone.0309837.s006.pdf]

S6 Fig. Publication bias assessment of Frailty prevalence

A) Funnel plot for Frailty prevalence

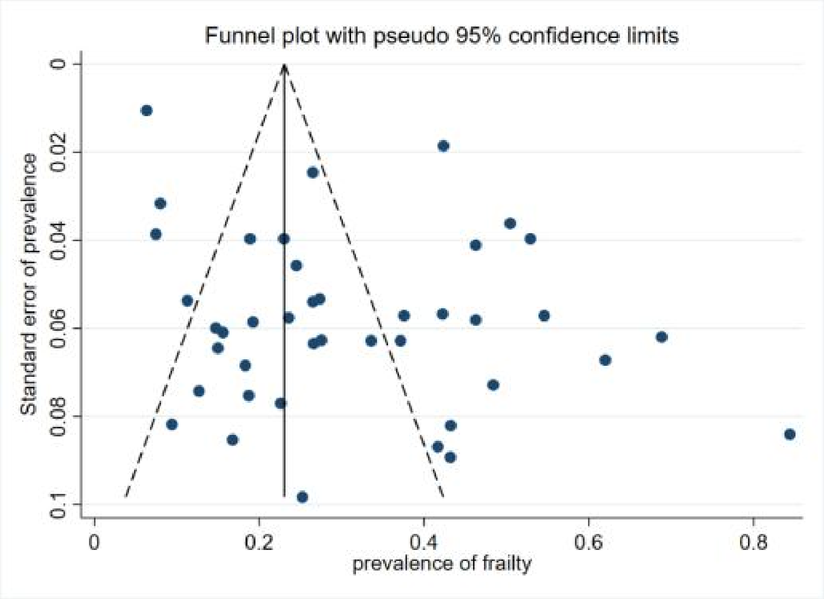

B) Egger's publication bias plot

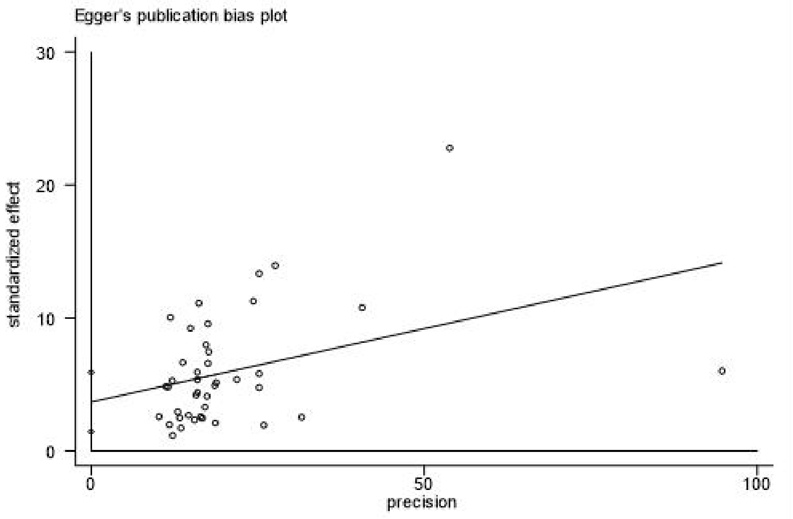

C) Results of Egger's regression test

Egger's test

Std_Eff	Coefficient	Std. err.	t	P>|t|	[95% conf.	interval]
slope	.1105604	.0438689	2.52	0.016	.0218271	.1992937
bias	3.668546	1.103199	3.33	0.002	1.437115	5.899977
