# Supplementary material for: Prevalence and risk factors of frailty in older adults with diabetes: A systematic review and meta-analysis
Source: PLoS One. 2024 Oct 31;19(10):e0309837. doi: 10.1371/journal.pone.0309837 (PMC11527323; doi:10.1371/journal.pone.0309837)
Supplement: S7 Fig — (PDF) [file pone.0309837.s007.pdf]

S7 Fig. Publication bias assessment of Pre-frailty prevalence

A) Funnel plot for Pre-frailty prevalence

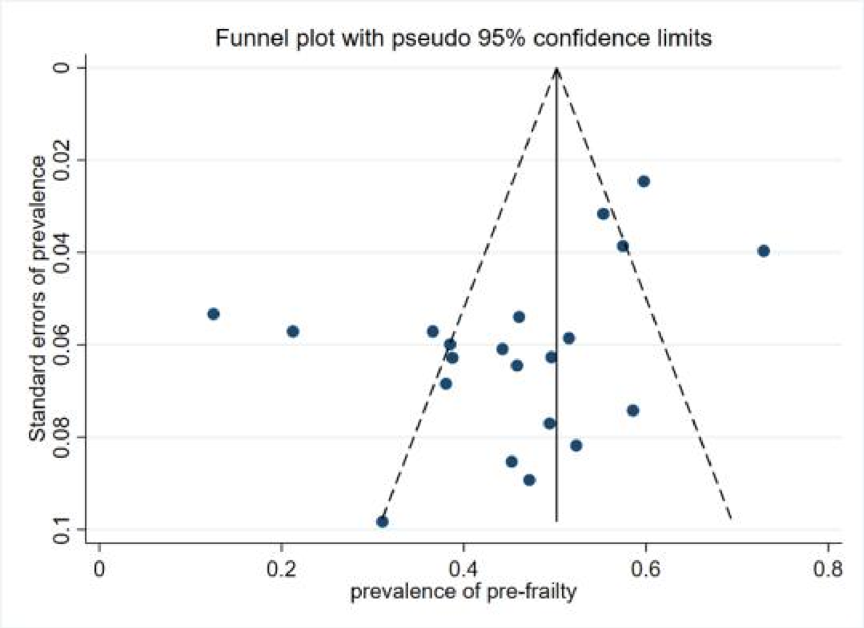

B) Egger's publication bias plot (Pre-frailty)

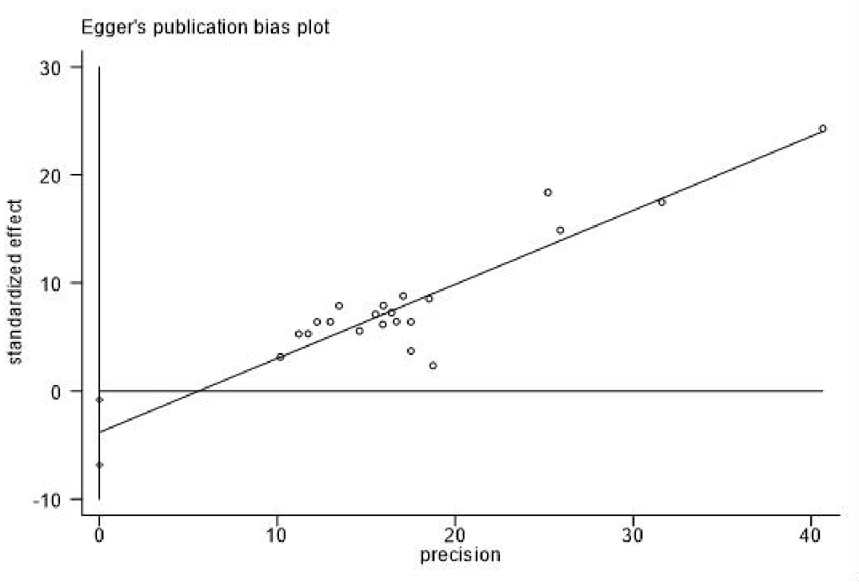

C) Results of Egger’s regression test(Pre-frailty)

Egger's test

Std_Eff	Coefficient	Std. err.	t	P>|t|	[95% conf.	interval]
slope	.6845513	.0741765	9.23	0.000	.5292981	.8398045
bias	-3.815663	1.441656	-2.65	0.016	-6.833084	-.7982413
