# Supplementary material for: Prevalence and risk factors of frailty in older adults with diabetes: A systematic review and meta-analysis
Source: PLoS One. 2024 Oct 31;19(10):e0309837. doi: 10.1371/journal.pone.0309837 (PMC11527323; doi:10.1371/journal.pone.0309837)
Supplement: S2 Table — (DOCX) [file pone.0309837.s009.docx]

**S2 Table.** PECOS model

| **PECOS model** | |
| --- | --- |
| **Participants:** | Individuals ≥60 years of age with diabetes. |
| **Exposure:** | Not applicable. |
| **Comparator/Control:** | Whether or not frailty occurs. |
| **Outcomes:** | Primary outcomes: Prevalence and risk factors of frailty in older patients with diabetes. |
|  | Secondary outcome: Prevalence of Pre-frailty in older patients with diabetes. |
| **Study design:** | cross-sectional /case-control / cohort studies. |
