# Supplementary material for: Prevalence and risk factors of frailty in older adults with diabetes: A systematic review and meta-analysis
Source: PLoS One. 2024 Oct 31;19(10):e0309837. doi: 10.1371/journal.pone.0309837 (PMC11527323; doi:10.1371/journal.pone.0309837)
Supplement: S3 Table — (DOCX) [file pone.0309837.s010.docx]

**S3 Table.** The search and screening strategy

| Electronic data | Results | search strategy (search performed on 15 January 2024) |
| --- | --- | --- |
| PubMed | 317 | #1 "Aged"[MeSH Terms] OR "Elderly"[Title/Abstract] OR "Frail Elderly"[Title/Abstract] OR "old people"[Title/Abstract] OR "elder*"[Title/Abstract] OR "older people"[Title/Abstract]  #2 "Diabetes Mellitus"[MeSH Terms] OR "Diabetes Insipidus"[Title/Abstract] OR "Diabetes Mellitus"[Title/Abstract] OR "Diabetes Insipidus"[Title/Abstract] OR "Glucose Intolerance"[Title/Abstract] OR Diabetes[Title/Abstract] OR Glycuresis[Title/Abstract] OR Diabetic[Title/Abstract] OR "type 2 diabetes"[Title/Abstract]  #3 "Frailty"[MeSH Terms] OR "Frailties"[Title/Abstract] OR "Frailness"[Title/Abstract] OR "frailty syndrome"[Title/Abstract] OR "Debility"[Title/Abstract] OR "Debilities"[Title/Abstract]  #4 "prevalence*"[Title/Abstract] OR "epidemiology"[Title/Abstract] OR "incidence*"[Title/Abstract] OR "morbidity"[Title/Abstract] OR factor*[Title/Abstract] OR "influencing factor*"[Title/Abstract] OR "relevant factor*"[Title/Abstract] OR "dangerous factor*"[Title/Abstract] OR factor*[Title/Abstract] OR risk[Title/Abstract]  #5 "cohort studies"[MeSH Terms] OR "case-control studies"[MeSH Terms] OR "comparative study"[pt] OR "risk factors"[MeSH Terms] OR "cohort"[tw] OR "compared"[tw] OR "groups"[tw] OR "case control"[tw] OR "multivariate"[tw]  #6 #1 AND #2 AND #3 AND #4 AND #5 |
| Embase | 2601 | #1 'aged'/exp OR 'frail elderly'/exp OR 'old people':ti,ab OR 'elder*':ti,ab OR 'older people':ti,ab  #2 'diabetes mellitus'/exp OR 'diabetes insipidus'/exp OR 'diabetes mellitus':ti,ab,kw OR 'diabetes insipidus':ti,ab,kw OR 'glucose intolerance':ti,ab,kw OR 'diabetes':ti,ab,kw OR 'glycuresis':ti,ab,kw OR 'diabetic':ti,ab,kw OR 'type 2 diabetes':ti,ab,kw  #3 'frailty'/exp OR 'frailty':ti,ab,kw OR 'frail*':ti,ab,kw OR 'frailty syndrome':ti,ab,kw OR 'Debility':ti,ab,kw OR 'Debilitie':ti,ab,kw  #4 'prevalence':ti,ab OR 'epidemiology':ti,ab OR 'incidence*':ti,ab OR 'morbidity':ti,ab OR 'factor*':ti,ab OR 'influencing factor*':ti,ab OR 'relevant factor*':ti,ab OR 'dangerous factor*':ti,ab OR 'factor*':ti,ab OR 'risk':ti,ab  #5 'clinical article'/exp OR 'controlled study'/exp OR 'major clinical study'/exp OR 'prospective study'/exp OR 'cohort analysis'/exp OR 'cohort':ti,ab OR 'compared':ti,ab OR 'groups':ti,ab OR 'case control':ti,ab OR 'multivariate':ti,ab  #6 #1 AND #2 AND #3 AND #4 AND #5 |
| Web of Science | 946 | #1 TS=(aged OR Elderly OR "Frail Elderly" OR "old people" OR "elder*" OR "older people" )  #2 TS=( "Diabetes Mellitus" OR "Diabetes Insipidus" OR "Glucose Intolerance" OR Diabetes OR Glycuresis OR Diabetic OR "type 2 diabetes" )  #3 TS=(Frailty OR Frailties OR Frailness OR "frailty syndrome" OR Debility OR Debilities) #4 AB=(prevalence* OR epidemiology OR incidence* OR morbidity OR factor* OR "influencing factor*" OR "relevant factor*" OR "dangerous factor*" OR factor* OR risk)  #5 AB=("cohort studies" OR "case-control studies" OR "comparative study" OR "risk factors" OR cohort OR compared OR groups OR case control OR multivariate)  #6 #1 AND #2 AND #3 AND #4 AND #5 |
| The Cochrane Library | 37 | #1 MeSH descriptor: [Aged] explode all trees  #2 (Elderly):ab,ti,kw OR (Frail Elderly):ab,ti,kw OR (old people):ab,ti,kw OR elder*:ab,ti,kw OR (older people):ab,ti,kw  #3 #1 OR #2  #4 MeSH descriptor: [Diabetes Mellitus] explode all trees  #5 MeSH descriptor: [Diabetes Insipidus] explode all trees  #6 (Glucose Intolerance):ab,ti,kw OR Diabetes:ab,ti,kw OR Glycuresis:ab,ti,kw OR Diabetic:ab,ti,kw OR (type 2 diabetes):ab,ti,kw  #7 #4 OR #5 OR #6  #8 MeSH descriptor: [Frailty] explode all trees  #9 Frailties:ab,ti,kw OR Frailness:ab,ti,kw OR (frailty syndrome):ab,ti,kw OR Debility:ab,ti,kw OR Debilities:ab,ti,kw  #10 #8 OR #9  #11 MeSH descriptor: [Prevalence] explode all trees  #12 epidemiology:ab,ti,kw OR incidence*:ab,ti,kw OR morbidity:ab,ti,kw OR factor*:ab,ti,kw OR (influencing factor*):ab,ti,kw OR (relevant factor*):ab,ti,kw OR (dangerous factor*):ab,ti,kw OR factor*:ab,ti,kw OR risk:ab,ti,kw  #13 #11 OR #12  #14 (cohort studies):ab,ti,kw OR (case-control studies):ab,ti,kw OR (comparative study):ab,ti,kw OR (risk factors):ab,ti,kw OR cohort:ab,ti,kw OR compared:ab,ti,kw OR groups:ab,ti,kw OR (case control):ab,ti,kw OR multivariate:ab,ti,kw  #15 #3 AND #7 AND #10 AND #13 AND #14 |
| CNKI | 174 | #1 主题=老年 OR 老年人  #2 主题=糖尿病 OR 2型糖尿病 OR 老年糖尿病 OR 老年2型糖尿病  #3 主题=衰弱 OR 衰弱综合征 OR 衰弱症  #4 主题=因素 OR 影响因素 OR 危险因素 OR患病率 OR 罹患率 OR 现患率 OR流行 OR 调查OR现况  #5 #1 AND #2 AND #3 AND #4 |
| CBM | 134 | #1 摘要=老年 OR 老年人  #2 摘要=糖尿病 OR 2型糖尿病 OR 老年糖尿病 OR 老年2型糖尿病  #3 摘要=衰弱 OR 衰弱综合征 OR 衰弱症  #4 摘要=因素 OR 影响因素 OR 危险因素OR患病率 OR 罹患率 OR 现患率 OR流行 OR 调查OR现况  #5 #1 AND #2 AND #3 AND #4 |
