# Supplementary material for: Prevalence and risk factors of frailty in older adults with diabetes: A systematic review and meta-analysis
Source: PLoS One. 2024 Oct 31;19(10):e0309837. doi: 10.1371/journal.pone.0309837 (PMC11527323; doi:10.1371/journal.pone.0309837)
Supplement: S4 Table — (DOCX) [file pone.0309837.s011.docx]

**S4 Table.** Excluded studies and reason (N = 57)

| **No.** | **Author/year** | **Title** | **Reason(s) for exclusion** |
| --- | --- | --- | --- |
| 1 | Crabtree et al., 2023 | All-cause mortality in individuals aged 65+ years with type 2 diabetes with and without frailty-clinical utility of electronic frailty index to individualise HbA1c targets. | Abstractonly |
| 2 | Almohid et al., 2020 | Clinical trial of metformin for frailty prevention: Is frailty associated with HBA1C at baseline? | Abstractonly |
| 3 | Balashova et al., 2022 | Elderly diabetes patients in long-term care facilities: A single center experience | Abstractonly |
| 4 | Nishimura et al., 2017 | Features of frailty and sarcopenia differ between sexes in elderly type 2 diabetes: A cross-sectional study | Abstractonly |
| 5 | Usoh et al., 2021 | Targeting deprescribing in type 2 diabetes mellitus using a pragmatic electronic health record measure of frailty | Abstractonly |
| 6 | Yates et al., 2019 | The prevalence of physical frailty in those with established type 2 diabetes | Abstractonly |
| 7 | Callahan et al., 2021 | Using the electronic health record for assessing deficit accumulation, to pragmatically identify candidates for de-prescribing in type 2 diabetes mellitus | Abstractonly |
| 8 | Priyadarshniet al., 2018 | Frailty and health care utilization among community-dwelling older patients with diabetes mellitus | Abstractonly |
| 9 | Kim al., 2018 | Frailty and risk of adverse events associated with dipeptidyl peptidase-4 inhibitors versus sulfonylureas in older adults with type 2 diabetes | Abstractonly |
| 10 | Callahan et al., 2022 | Frailty, glycemic control, and patterns of healthcare utilization among older adults with type 2 diabetes mellitus | Abstractonly |
| 11 | Madden et al., 2021 | Glycemic control and frailty in older adults with type 2 diabetes | Abstractonly |
| 12 | Valencia et al., 2018 | History of falls in the previous year predicts frailty among community-dwelling older patients with diabetes mellitus | Abstractonly |

| **No.** | **Author/year** | **Title** | **Reason(s) for exclusion** |
| --- | --- | --- | --- |
| 13 | Guerra et al., 2019 | Is metformin associated with frailty in older veterans with diabetes? | Abstractonly |
| 14 | Wongviriyawong et al., 2015 | Optimum level of glycaemic control for the lowest mortality in very old adults with type 2 diabetes mellitus | Abstractonly |
| 15 | Sable-Morita et al., 2017 | Relationship between hearing impairment and frailty in older patients with diabetes mellitus | Abstractonly |
| 16 | Nguyen et al., 2019 | Relationship between sarcopenia and frailty on pre-diabetic and diabetic older patients | Abstractonly |
| 17 | Crabtree et al., 2023 | Risk of hospital admission in individuals aged 65 years with type 2 diabetes with and without frailty: The role of electronic frailty index to individualise HbA1c targets | Abstractonly |
| 18 | Chang et al., 2023 | Short term structured, multimodal, interdisciplinary, home-based, self-help program is effective in improving frailty status among elderly T2DM patients | Abstractonly |
| 19 | Nishimura et al., 2016 | Specific factors associated with frailty in elderly type 2 diabetes patients: A cross-sectional study | Abstractonly |
| 20 | Palabiyik et al., 2022 | assessment of the relationship between hypoglycemia and frailty in elderly people with diabetes | Abstractonly |
| 21 | Song et al., 2022 | Study on influencing factors of frailty in elderly patients with type II diabetes | Article retracted |
| 22 | Cristina et al., 2018 | Frailty in older adults with type 2 diabetes mellitus and its relation with glucemic control, lipid profile, blood pressure, balance, disability grade and nutritional status | Not in English or Chinese |
| 23 | Cunha et al., 2023 | Frailty Syndrome and sarcopenia in older adults with and without type 2 diabetes mellitus in the municipality of Sinop, Mato Grosso: an epidemiological study | Not in English or Chinese |

| **No.** | **Author/year** | **Title** | **Reason(s) for exclusion** |
| --- | --- | --- | --- |
| 24 | Colorado et al., 2020 | Nutritional status and its association with fragility in the elderly with type 2 diabetes mellitus | Not in English or Chinese |
| 25 | Kirkwood et al., 2019 | Frailty status and gait parameters of older women with type 2 diabetes. | Sample size<100 |
| 26 | Simó-Servat et al., 2023 | Role of muscle ultrasound for the study of frailty in elderly patients with diabetes: A pilot study | Wrong outcomes: prevalence of frailty data cannot be extracted |
| 27 | Deborah et al., 2018 | Frailty risk in hospitalised older adults with and without diabetes mellitus. | Wrong population |
| 28 | Olson et al., 2023 | Psychological resilience in older adults with type 2 diabetes from the Look AHEAD Trial | Wrong population |
| 29 | Espinoza et al., 2022 | The association of prior intensive lifestyle intervention and diabetes support and education with frailty prevalence at long-term follow-up in the action for health in diabetes extension study | Wrong population |
| 30 | Chi et al., 2023 | The impact of glucose-lowering strategy on the risk of increasing frailty severity among 49,519 patients with diabetes mellitus: A longitudinal cohort study | Wrong population |
| 31 | Chhetri et al., 2017 | The prevalence and incidence of frailty in Pre-diabetic and diabetic community-dwelling older population: results from Beijing longitudinal study of aging II (BLSA-II) | Wrong population |
| 32 | Nie et al., 2017 | Frailty syndromes and geriatric diabetes | Wrong study design: a review |
| 33 | Bahat et al., 2022 | Management of type 2 diabetes mellitus in older adults: eight case studies with focus SGLT-2 inhibitors and metformin | Wrong study design: a case study |
| 34 | Chung et al., 2021 | Daytime glycemic variability and frailty in older patients with diabetes: a pilot study using continuous glucose monitoring. | Studies assessed as highrisk of bias |
| 35 | Mangé et al., 2021 | Diabetes and frail older patients: Glycemic control and prescription profile in real life | Studies assessed as highrisk of bias |
| 36 | Li et al., 2018 | Frailty and health care use among community-dwelling older adults with diabetes: a population-based study | Studies assessed as highrisk of bias |
| **No.** | **Author/year** | **Title** | Studies assessed as highrisk of bias |
| 37 | Alabadi et al., 2021 | Frailty is associated with oxidative stress in older patients with type 2 diabetes. | Studies assessed as highrisk of bias |
| 38 | Yanagita et al., 2020 | Low serum albumin, aspartate aminotransferase, and body mass are risk factors for frailty in elderly people with diabetes-a cross-sectional study. | Studies assessed as highrisk of bias |
| 39 | Ganidagli et al., 2023 | Poor sleep quality is an overlooked risk for geriatric syndromes in older adults with type 2 diabetes mellitus | Studies assessed as highrisk of bias |
| 40 | Lee et al., 2022 | Predictors associated with prefrailty in older Taiwanese individuals with type 2 diabetes. | Studies assessed as highrisk of bias |
| 41 | Sato et al., 2023 | The cross-sectional area of erector spinae muscle and the liver-to-spleen ratio are associated with frailty in older patients with diabetes: a cross-sectional study. | Studies assessed as highrisk of bias |
| 42 | García-de-Alba-García et al., 2020 | The status of frailty in poor older adults with type 2 diabetes mellitus or hypertension: the case of Mexico | Studies assessed as highrisk of bias |
| 43 | Callahan et al., 2022 | Using an Electronic Health Record and Deficit Accumulation to Pragmatically Identify Candidates for Optimal Prescribing in Patients With Type 2 Diabetes | Studies assessed as highrisk of bias |
| 44 | İDi̇Z et al., 2021 | WHAT ARE THE FACTORS AFFECTING THE FRAILTY OF ELDERLY PEOPLE WITH TYPE 2 DIABETES? | Studies assessed as highrisk of bias |
| 45 | Hou et al., 2020 | Analysis of risk factors of frailty in patients with type 2 diabetes mellitus | Studies assessed as highrisk of bias |
| 46 | Zhang et al., 2022 | Analysis of the status of frailty and influencing factors in elderly diabetic patients of different genders | Studies assessed as highrisk of bias |
| 47 | Bao et al., 2022 | Analysis of influencing factors of frailty based on geriatric comprehensive assessment in the elderly patients with type 2 diabetes mellitus | Studies assessed as highrisk of bias |

| **No.** | **Author/year** | **Title** | **Reason(s) for exclusion** |
| --- | --- | --- | --- |
| 48 | Zhang et al., 2020 | Correlation between glucose and lipid metabolism indexes and degree of frailty in elderly patients with type 2 diabetes mellitus | Studies assessed as highrisk of bias |
| 49 | He et al., 2022 | Analysis of risk factors for comorbid frailty in elderly patients with type 2 diabetes mellitus | Studies assessed as highrisk of bias |
| 50 | Zhang et al., 2022 | Analysis of the current situation and risk factors of frailty in elderly patients with type 2 diabetes | Studies assessed as highrisk of bias |
| 51 | Xiu et al., 2021 | Risk factors of frailty in the elderly with type 2 diabetes mellitus | Studies assessed as highrisk of bias |
| 52 | Si et al., 2023 | Influential Factors for Frailty in Older Patients with Type 2 Diabetes Mellitus | Studies assessed as highrisk of bias |
| 53 | Han et al., 2021 | Influential Factors and Countermeasures for Frailty in Older Patients with Type 2 Diabetes Mellitus | Studies assessed as highrisk of bias |
| 54 | Chen et al., 2019 | Analysis of frailty and its influencing factors in inpatients with type 2 diabetes | Studies assessed as highrisk of bias |
| 55 | Ling et al., 2021 | Related factors of frailty in elderly inpatients with type 2 diabetes mellitus | Studies assessed as highrisk of bias |
| 56 | Meng,2021 | Correlation between depressive status and frailty and glycaemic control in elderly patients with type 2 diabetes mellitus | Studies assessed as highrisk of bias |
| 57 | Zhang et al., 2022 | Analysis of frailty and factors affecting frailty in elderly patients first diagnosed with diabetes mellitus | Studies assessed as highrisk of bias |
