# Supplementary material for: Prevalence and risk factors of frailty in older adults with diabetes: A systematic review and meta-analysis
Source: PLoS One. 2024 Oct 31;19(10):e0309837. doi: 10.1371/journal.pone.0309837 (PMC11527323; doi:10.1371/journal.pone.0309837)
Supplement: S6 Table — (DOCX) [file pone.0309837.s013.docx]

**S6 Table.** Assessment of the quality of cross-sectional studies based on the criteria recommended by the US Agency for Healthcare Quality and Research (AHRQ).

| Study | Q1 | Q2 | Q3 | Q4 | Q5 | Q6 | Q7 | Q8 | Q9 | Q10 | Q11 | Quality |
| --- | --- | --- | --- | --- | --- | --- | --- | --- | --- | --- | --- | --- |
| Guo 2019 | Yes | Yes | Yes | Unclear | Unclear | No | No | No | No | Yes | No | moderate |
| Guo 2018 | Yes | Yes | Yes | Unclear | Unclear | Yes | No | No | No | Yes | No | moderate |
| Sun 2024 | Yes | Yes | Yes | Unclear | Unclear | No | No | No | Yes | Yes | No | moderate |
| Cheng 2020 | Yes | Yes | Yes | Unclear | Unclear | Yes | No | No | No | Yes | No | moderate |
| Liu 2023 | Yes | Yes | Yes | Unclear | Unclear | No | Yes | No | No | No | No | moderate |
| Ge X 2020 | Yes | Yes | Yes | Unclear | Unclear | No | No | No | No | Yes | No | moderate |
| Jia 2019 | Yes | Yes | Yes | Unclear | Unclear | No | No | No | No | Yes | No | moderate |
| Chen 2019 | Yes | Yes | Yes | Unclear | Unclear | Yes | No | No | No | Yes | No | moderate |
| Sun 2020 | Yes | Yes | Yes | Unclear | Unclear | No | Yes | No | No | No | No | moderate |
| Wu 2021 | Yes | No | Yes | Yes | Unclear | No | No | No | No | Yes | No | moderate |
| Ge Q 2020 | Yes | Yes | Yes | Yes | Unclear | Yes | No | No | No | Yes | No | moderate |
| Li 2022 | Yes | Yes | Yes | Yes | Unclear | No | No | No | No | Yes | No | moderate |
| Deng 2020 | Yes | Yes | Yes | Yes | Unclear | No | No | No | No | Yes | No | moderate |
| Zhao 2023 | Yes | No | Yes | Unclear | Unclear | No | No | No | Yes | Yes | No | moderate |
| Wang&Li 2023 | Yes | Yes | Yes | Yes | Unclear | No | No | No | No | Yes | No | moderate |
| Sun 2021 | Yes | Yes | Yes | Unclear | Unclear | No | Yes | No | No | No | No | moderate |
| Hayakawa 2021 | Yes | Unclear | Yes | No | Yes | No | Yes | No | No | Yes | No | moderate |
| Kong 2021 | Yes | Yes | Yes | Yes | Yes | Yes | No | No | No | Yes | No | moderate |
| Wang&Wang 2023 | Yes | Yes | Yes | Unclear | No | No | No | No | No | Yes | Yes | moderate |
| Lin 2022 | Yes | Yes | Yes | Unclear | No | No | Yes | No | No | No | No | moderate |
| Nishimura 2019 | Yes | Yes | Yes | Unclear | Yes | No | Yes | No | No | No | No | moderate |
| Kang 2021 | Yes | Yes | Yes | Yes | Unclear | Yes | No | No | No | Yes | No | moderate |
| Cacciatore 2013 | Yes | Unclear | Yes | Unclear | Yes | Yes | No | No | No | Yes | Yes | moderate |
| MacKenzie 2020 | Yes | Unclear | Yes | Unclear | Yes | Yes | No | No | No | No | No | moderate |
| Nguyen 2020 | Yes | Yes | Yes | Unclear | Yes | No | No | No | No | No | No | moderate |
| Lima Filho 2020 | Yes | Yes | Yes | Unclear | Yes | Yes | Yes | No | No | No | No | moderate |
| Muszalik 2022 | Yes | Yes | Yes | Unclear | Unclear | Yes | No | No | No | No | No | moderate |
| Xiu 2020 | Yes | Yes | Yes | Unclear | Unclear | Yes | Yes | No | No | No | No | moderate |
| Bąk 2021 | Yes | Yes | Yes | Unclear | Unclear | Yes | Yes | No | No | No | No | moderate |

Studies scoring 0 to 3 are considered low quality, 4 to 7 indicates moderate quality, and 8 to 11 are considered high quality.
