# Supplementary material for: Prevalence and risk factors of frailty in older adults with diabetes: A systematic review and meta-analysis
Source: PLoS One. 2024 Oct 31;19(10):e0309837. doi: 10.1371/journal.pone.0309837 (PMC11527323; doi:10.1371/journal.pone.0309837)
Supplement: S7 Table — (DOCX) [file pone.0309837.s014.docx]

**S7 Table.** Quality of cohort studies based on the Newcastle–Ottawa scale.

| Studies | Selection  (0–4 stars) | | | | Comparability  (0–2 stars) | Exposure  (0–3 stars) | | | Total NOS  score (0–9) | Quality |
| --- | --- | --- | --- | --- | --- | --- | --- | --- | --- | --- |
|  | Q1 | Q2 | Q3 | Q4 | Q5 | Q6 | Q7 | Q8 |  |  |
| Qin 2023 | ⁕ | ⁕ | ⁕ |  | ⁕ |  | ⁕ |  | 5 | moderate |
| Zaslavsky 2016 | ⁕ | ⁕ | ⁕ | ⁕ | ⁕ |  | ⁕ |  | 6 | moderate |
| Yanagita 2018 | ⁕ | ⁕ | ⁕ |  |  | ⁕ |  |  | 4 | moderate |
| Lopez-Garcia 2018 | ⁕ | ⁕ | ⁕ |  | ⁕⁕ | ⁕ | ⁕ | ⁕ | 8 | High |
| Sable Morita 2021 | ⁕ | ⁕ | ⁕ | ⁕ | ⁕ |  |  | ⁕ | 6 | moderate |
| Aguayo 2019 | ⁕ | ⁕ | ⁕ |  | ⁕ | ⁕ | ⁕ |  | 6 | moderate |
| Ferri-Guerra 2020 | ⁕ | ⁕ | ⁕ |  | ⁕⁕ | ⁕ | ⁕ | ⁕ | 8 | High |
| García-Esquinas 2015 | ⁕ | ⁕ | ⁕ | ⁕ | ⁕ | ⁕ | ⁕ | ⁕ | 8 | High |
| Kitamura 2019 | ⁕ | ⁕ | ⁕ |  | ⁕ | ⁕ | ⁕ | ⁕ | 7 | moderate |
| Hubbard 2011 | ⁕ | ⁕ | ⁕ |  | ⁕ |  | ⁕ | ⁕ | 6 | moderate |
|  |  |  |  |  |  |  |  |  |  |  |
| Q1: Representativeness of the exposed cohort | | | | | | |  |  |  |  |
| Q2: Selection of the non-exposed cohort | | | | | | |  |  |  |  |
| Q3: Ascertainment of exposure | | | | | | |  |  |  |  |
| Q4: Demonstration that outcome of interest was not present at the start of the study | | | | | | | | | |  |
| Q5: Comparability of cohorts on the basis of the design or analysis | | | | | | |  |  |  |  |
| Q6: Assessment of outcome | | | | | | |  |  |  |  |
| Q7: Was followed up long enough for outcomes to occur | | | | | | |  |  |  |  |
| Q8: Adequacy of follow-up of cohorts | | | | | | |  |  |  |  |
| “⁕” represents one point | | | | | | |  |  |  |  |

High quality was considered when the assigned score was ≥8, moderate quality when the score was between four and seven and low quality when the score was ≤3.
