# Supplementary material for: Prevalence and risk factors of frailty in older adults with diabetes: A systematic review and meta-analysis
Source: PLoS One. 2024 Oct 31;19(10):e0309837. doi: 10.1371/journal.pone.0309837 (PMC11527323; doi:10.1371/journal.pone.0309837)
Supplement: Attachment — Submitted filename: Response to Reviewers.docx [file pone.0309837.s015.docx]

Dear Editor and Reviewers:

Thank you for your decision and constructive comments on our manuscript. We have carefully considered the suggestions of the editor and reviewers and made several changes. We have made our best efforts to improve the manuscript. The comments are laid out below in italicized font and specific concerns have been numbered. Our response is given in normal font and changes/additions to the manuscript are given in the yellow text.

Academic Editor:

*1)Please ensure that your manuscript meets PLOS ONE's style requirements, including those for file naming.*

Thanks for your reminder. We have modified the reference style of the manuscript according to PLOS ONE's style requirements. In addition, we have added the title of the figures.

*2)We noticed you have some minor occurrence of overlapping text with the following previous publication(s)*

We appreciate your thorough review. We have revised the overlapping text in the manuscript. (P2 Line26-28, P3 Line67-71, P3 Line82-83 and P18 Line53-54 marked in yellow in the revised paper)

*3)Please amend your authorship list in your manuscript file to include author Yuanhong Sun.*

Thanks for your reminder. The author Yuanhong Sun will be added to the list of authors via the submission system. (P1 Line4 marked in yellow in the revised paper)

*4)Please include captions for your Supporting Information files at the end of your manuscript, and update any in-text citations to match accordingly.*

We appreciate your attention to detail. We have added the captions of the supporting information and cited them in the corresponding location in the body of the text.

*5)Please review your reference list to ensure that it is complete and correct.*

The reference list has been checked and modified.

Reviewer#1:

*The frailty is actually considered a multidimensional frailty and 4 different domains should be considered (physical, mental, nutritional and social). Please discuss this matter in the revised form of the manuscript.*

We sincerely appreciate your valuable comments. We have addressed this issue in the revised manuscript. (P18 Line63-90 marked in yellow in the revised paper)

Reviewer#2:

*Data suffer from the different instrument used in literature to identify frailty status. But the analysis was conducted in order to analyze these differences.*

We sincerely appreciate the valuable comments. We provide a detailed discussion of these differences through subgroup analyses and meta-regression. (P20 Line114-125 marked in yellow in the revised paper)

Thanks again for your professional review work on our article. If you have any queries, please don’t hesitate to contact me at the address below.

Yours sincerely,

Li Liao

School of Nursing, University of South China, Hengyang, Hunan Province, China

liaoli@usc.edu.cn
